# Protocol for a scoping review on rehabilitation among individuals with traumatic brain injury who intersect with the criminal justice system

**DOI:** 10.1371/journal.pone.0269696

**Published:** 2022-06-30

**Authors:** Vincy Chan, Maria Jennifer Estrella, Zacharie Beaulieu-Dearman, Jessica Babineau, Angela Colantonio

**Affiliations:** 1 KITE-Toronto Rehabilitation Institute, University Health Network, Toronto, Ontario, Canada; 2 Institute of Health Policy, Management and Evaluation, University of Toronto, Toronto, Ontario, Canada; 3 Rehabilitation Sciences Institute, University of Toronto, Toronto, Ontario, Canada; 4 Department of Occupational Science & Occupational Therapy, University of Toronto, Toronto, Ontario, Canada; 5 Department of Health Sciences, Lakehead University, Thunder Bay, Ontario, Canada; 6 Library & Information Services, University Health Network, Toronto, Ontario, Canada; 7 The Institute for Education Research, University Health Network, Toronto, Ontario, Canada; UNITED STATES

## Abstract

Traumatic brain injury (TBI), a leading cause of both death and disability worldwide, is highly prevalent among individuals who intersect with the criminal justice system. TBI is associated with increased behavioural, psychological, or negative outcomes, such as higher rates of mental health problems, aggression, and violent offending that may lead to negative interactions with the criminal justice system, reincarceration, and recidivism. Although rehabilitation is often recommended and holds promise in addressing TBI-related impairments, there is currently a paucity of reviews on rehabilitation for individuals with TBI who intersect with the criminal justice system (CJS). Concurrently, to the best of our knowledge, there is currently no review that considers rehabilitation among individuals with TBI who intersect with all parts of the CJS (i.e., policing, courts, corrections, and parole). This protocol is for a scoping review to address the above gaps, specifically, to identify the types of rehabilitation interventions and/or programs available to, or used by, individuals with TBI who intersect with all parts of the CJS. Primary research articles that meet pre-defined inclusion criteria will be identified from electronic databases (MEDLINE® ALL, Embase and Embase Classic, Cochrane CENTRAL Register of Clinical Trials, CINAHL, APA PsycINFO, Applied Social Sciences Index and Abstracts, Criminal Justice Abstracts, Nursing and Allied Health, and Dissertation and These Global), reference lists of included articles, and scoping or systematic reviews. Grey literature will also be searched to identify non-peer-reviewed reports. Retrieved articles will be screened by two reviewers and any disagreements will be resolved by a third reviewer. Data will be summarized quantitatively and analyzed using content analytic techniques. Intersecting identities will be charted and considered in the analysis. Stakeholders will be engaged to obtain feedback on preliminary results and the implications of findings. The scoping review will summarize the current state of rehabilitation available to, or used by, individuals with TBI who intersect with all parts of the CJS to (a) inform opportunities to integrate rehabilitation in the criminal justice system for diverse individuals and (b) identify opportunities for future research.

## Introduction

Traumatic brain injury (TBI) is a leading cause of disability and death worldwide [1]. It is estimated to affect 64 to 74 million individuals annually [1] and is disproportionately prevalent among individuals who intersect with the criminal justice system (CJS). Two meta-analyses reported a lifetime prevalence of TBI of 51% [2] and 60% [3] in incarcerated populations while systematic reviews reported prevalence ranging from 9.7% to 100% across all ages [4], 16.5% to 72.1% among youths [5], 25% to 85% among adults [6]. A systematic review specifically on female incarcerated individuals found that the prevalence of TBI ranged from 28% to 49% among youths and 19% to 95% among adults [7]. Importantly, the prevalence of TBI in incarcerated populations has been reported to be significantly higher than that of the general population, with a meta-analysis reporting 2.0% to 38.5% [2] and a systematic review of youths reporting 4.7% to 35.0% [5].

While no causal relationship has been established between TBI and involvement with the CJS, evidence suggests a bidirectional relationship. Individuals who engage in risk-taking behaviours are likely to engage in actions that may lead to offending and injury, including TBI [5, 8, 9]. On the other hand, a history of TBI is associated with cognitive deficits and mental health challenges, drug and alcohol use, increased rates of violent behavior, earlier age of incarceration [9], and serious disciplinary charges [10]. Furthermore, specifically among incarcerated females, those who experienced a TBI were more likely to have experienced adverse early life experiences, including physical and sexual abuse [13], a history of violent offences, and problematic substance use [8] compared to incarcerated females without a history of TBI. These challenges experienced by individuals with a history of TBI can be long-lasting and can impact their interactions with the CJS [11], including increased likelihood of reoffending compared to individuals without a TBI [12–15]. For example, TBI may result in memory challenges (e.g., forgetting details of an event, conversation, or appointments) [16], difficulties in expressing thoughts and understanding the language used in court and criminal proceedings [17], and behaviours that are often viewed as defiant or uncooperative (e.g., not being able to focus or respond to directions, not understanding or remembering rules and inadvertently violating them, or slow verbal and physical responses that may be interpreted as uncooperative behaviour) [11]. If unaddressed, TBI and sequalae of TBI may lead to a cycle of reincarceration and recidivism [14, 15, 18, 19].

Rehabilitation, as defined by the World Health Organization (WHO), is “a set of interventions designed to optimize functioning and reduce disability in individuals with health conditions in interaction with their environment.” [20] Rehabilitation after TBI is considered an integral part of post-injury care and holds the potential to promote recovery and address disability associated with TBI [20]. According to existing clinical practice guidelines for TBI, rehabilitation after TBI encompasses assessment and treatment of brain injury sequelae, such as motor, cognitive, communication, and psychosocial challenges [21]. Evidence suggests that rehabilitation after TBI can reduce complications and comorbidities, improve return to work and community integration, and reduce overall healthcare cost [22–26]. However, despite its reported importance and benefits, most reviews on TBI in the CJS to date focus on identifying the prevalence of TBI, not rehabilitation, and/or are limited to the corrections setting [2–7, 27]. As such, it is unclear whether the current literature on rehabilitation after TBI considers all components of the CJS and concurrently, whether the literature on rehabilitation provided in correctional settings considers TBI-specific concerns.

This is an important research and knowledge gap. Given that individuals with TBI are over-represented in the incarcerated population [2], it can be expected that a large number of individuals with TBI will also intersect with the policing, court, and parole systems. Additionally, existing correctional rehabilitation frameworks and programs may not always be effective, due to the lack of specificity in addressing TBI-related impairments. For example, the cognitive skills framework predominately used in American and European correctional remediation programs focuses on developing cognitive skills that refer to prosocial thoughts, attitudes, and actions [22]. Rehabilitation interventions that utilize this framework target maladaptive thoughts and beliefs to improve emotional well-being and behaviour with the goal of reducing recidivism [22]. However, it is not clear whether rehabilitation interventions that utilize this framework target executive cognitive functioning, a set of cognitive abilities often affected by TBI that may present as deficits in planning, concept formation, mental flexibility, aspects of attention and awareness, and purposeful behavior [22, 28]. Such deficits, in addition to other cognitive and emotional challenges experienced by individuals with TBI, must be considered in rehabilitation after TBI, as they can interfere with an individual’s ability to benefit from rehabilitation interventions, particularly if the intervention requires learning new skills [18, 22]. Identifying existing rehabilitation for individuals with TBI who intersect with all stages of the CJS is critical to building a foundation of research that ensures rehabilitation is available to and tailored to the specific needs and challenges of individuals with TBI who intersect with the CJS.

This protocol is for a scoping review that aims to explore the types of rehabilitation interventions and/or programs available to, or used by, individuals with TBI who intersect with the CJS. In particular, all parts of the CJS–policing, courts, corrections, and parole [29–31]–will be explored, to address the paucity of reviews on TBI outside of the corrections system. Specifically, we will be exploring interventions and/or programs used by individuals with TBI who are involved with police interactions and arrests, appear in court, are incarcerated or have experienced incarceration, or are on probation or parole. This protocol also explicitly outlines the identification of findings across sex, gender, intersecting identities (e.g., race, ethnicity, disability), and experiences with violence and homelessness in the charting and analysis stages of the review to understand existing rehabilitation for diverse individuals. Findings from the scoping review will comprehensively summarize the current state of rehabilitation among individuals with TBI who intersect with all parts of the CJS to (a) inform opportunities to integrate rehabilitation in the CJS and (b) identify opportunities for future research.

### Methods and analysis

We will follow the scoping review methodology framework introduced by Arksey and O’Malley [32] and expanded by Levac et al. [33]. This framework consists of the following six stages: identifying the research question; identifying relevant studies; study selection; charting the data; collating, summarizing, and reporting results; and consultation [33]. The reporting of the scoping review will be guided by the Preferred Reporting Items for Systematic reviews and Meta-Analyses extension for Scoping Reviews (PRISMA-ScR) [34].

### Identifying the research question

The scoping review will answer the research question: “What are the types of rehabilitation interventions and/or programs available to, or used by, individuals with TBI who intersect with the CJS?” The parameters and definitions in Table 1 will guide our scoping review and search strategy, as well as the study selection, charting of data, and reporting of findings.

**Table 1:** Parameters and associated definitions for rehabilitation and the criminal justice system.

Concept	Parameter	Definition
Traumatic Brain Injury (TBI)	Definition of TBI by the Demographics and Clinical Assessment Working Group of the International and Interagency Initiative toward Common Data Elements for Research on Traumatic Brain Injury and Psychological Health [35]	“An alteration in brain function, or other evidence of brain pathology, caused by an external force.”
Rehabilitation	World Health Organization’s definition of rehabilitation [20]	“A set of interventions designed to optimize functioning and reduce disability in individuals with health conditions in interaction with their environment”
	Healthcare providers/professional disciplines identified in evidence-based clinical practice guidelines for rehabilitation [21, 36]	• Neuropsychologist and psychometrist • Nurse • Nutritionist • Occupational therapist • Physician and/or physiatrist • Physiotherapist • Psychologist with expertise in behavioural therapy • Rehabilitation support personnel • Social worker • Speech-language pathologists • Therapeutic recreationist
Criminal justice system	Parts of the criminal justice system described by Correctional Service Canada [29], United Kingdom [30], and United States [31]	• Policing: Apprehends suspects • Courts: Decides on charges, prosecutes charges, determines sentence • Corrections: Administers the sentence • Parole: Determines if the individual qualifies for early release to the community and the attendant conditions thereof

### Identifying relevant studies

The search strategy presented in this protocol was developed with an Information Specialist (JB) and team members with research and subject-matter expertise relevant to rehabilitation, TBI, and the CJS (see S1 File). Sections of the strategy were also informed by comprehensive search strategies applied to previous knowledge syntheses [37, 38]. Specifically, the search strategy was developed for the MEDLINE® ALL (in Ovid, including Epub Ahead of Print, In-Process & Other Non-Indexed Citations, Ovid MEDLINE(R) Daily) database and will be translated to: Embase and Embase Classic (Ovid), Cochrane CENTRAL Register of Clinical Trials (Ovid), CINAHL (EBSCO), APA PsycINFO (Ovid), Applied Social Sciences Index and Abstracts (Proquest), Criminal Justice Abstracts (EBSCO), Nursing and Allied Health (Proquest), and Dissertation and These Global (Proquest).

The following concepts were developed to form the search strategy:

- Criminal justice system
- Rehabilitation
- TBI or cognitive impairment

The final search strategy structure, (A + B + C), will be used to search each database to identify peer-reviewed primary research and review articles. Grey literature, operationalized as reports from relevant brain injury, CJS, and rehabilitation organizations, will be identified from the organizations’ websites (see S1 File) and through consultation with stakeholders of our research (see Consultation section). We will also search the reference lists of included primary research articles, scoping or systematic reviews, and grey literature. Search strategies will be limited to human populations, when possible. No language or date limits will be placed on search strategies. Reporting of the search strategy will follow PRISMA-S extension recommendations [39].

### Study selection

The following inclusion criteria for our scoping review will apply to research articles, grey literature, and scoping or systematic reviews:

1. Describe or document interventions/treatments/programs designed to optimize functioning and reduce disability in individuals with health conditions in interaction with their environment or describe and/or document rehabilitation services provided by healthcare providers/professional disciplines, as defined in Table 1
2. Include individuals who have intersected with the CJS, as defined in Table 1;
3. Include individuals with TBI (identified through diagnosis, screening, or self-report), with TBI as defined in Table 1; and
4. Report primary research findings.

The exclusion criteria are as follows:

1. Books and conference proceedings; or
2. Articles, grey literature, and reviews that are narrative, commentaries, or describe a theory or framework without reporting primary research findings; or
3. Articles that describe a sample including brain injury (e.g., acquired brain injury) or individuals experiencing cognitive impairment without specific mention of TBI.

Relevant studies retrieved using the above search strategy will be imported into EndNote X8.2 [40] for reference management and Covidence [41] for deduplication and study selection. All articles will be screened independently by two reviewers according to the aforementioned inclusion and exclusion criteria. At the title and abstract screen, articles that mention brain injury or individuals experiencing cognitive impairment without specific mention of TBI will also be considered for the full-text screen to confirm the study includes individuals with TBI. At the full-text screen, scoping and systematic reviews that meet the inclusion criteria will be further assessed by retrieving the primary research articles included in these reviews. Only primary research articles that meet the inclusion criteria will be included in this scoping review.

Before the formal screening process begins, pilot screening of 20 titles and abstracts will be conducted, until a minimum 80% agreement using the kappa statistic is achieved between the reviewers. At the full-text review, pilot screening of 10% of the full-text articles will be conducted until a minimum of 80% agreement using the kappa statistic is achieved between the reviewers. Non-English language abstracts will be assessed using the published English abstract; full text articles that meet eligibility criteria will be screened using the English full-text translation, Google Translate, DeepL Translate, or reviewers with knowledge of the language. Disagreement between the reviewers during either study selection process will be resolved by consultation with a third reviewer. The study selection process will be presented using the Preferred Reporting Items for Systematic Reviews and Meta Analyses flow chart [34].

### Charting the data

Table 2 presents the charting table for the scoping review, which will be iteratively improved during the research process, as recommended by Levac et al. [33]. One reviewer will independently complete the charting table for each study and the completed table will then be independently reviewed by a second reviewer. As in the study selection stage, a random sample of five articles will be charted until a minimum of 80% agreement is achieved between the reviewers. Discrepancies in charting the data will be resolved by consultation with a third reviewer. If any of the data items are not noted in the retrieved articles, it will be recorded as “not reported”.

**Table 2:** Charting table.

Data Item		Description
Study characteristics	Author (Year of publication)	
	Country of study	
	Type of article	Note if the article was a peer-reviewed publication or grey literature
	Study design	Specify if the study was quantitative, qualitative, or mixed methods and describe the study design
	Objective	State the objective of the study
Study sample	TBI	Specify the definition of TBI and how TBI was determined (e.g., screening tool, diagnostic criteria)
		Specify the injury severity, time since injury, timing of TBI relative to CJS interaction (e.g., whether TBI predated CJS involvement, if the individual is currently intersecting with the CJS), and the sample (N, %) of individuals with TBI
	Intersection with the CJS	Specify the nature of CJS intersection (policing, courts, corrections, and/or parole)
		Specify the sample (N, %) of individuals with TBI who intersect with the CJS
	Age	Specify participants’ age at the time of the study, at the time of TBI, and at the time of CJS intersection
	Sex/Gender	Specify if Sex- and Gender-Based Analysis Plus (SGBA+) was considered in the study design [42]
		Note if and/or how sex and gender were defined in the study, including data on sexual orientation and gender identity.
		Specify the participants’ sex and/or gender (N, %)
	Sociodemographic	Specify sociodemographic characteristics of the sample (e.g., race, ethnicity, religion, disability, geography, culture, income, education)
		Note if/describe how the sample of also experienced homelessness and/or violence, including intimate partner violence
		Note if/describe how the article acknowledged and/or accounted for intersecting social identities and/or vulnerabilities
Rehabilitation	Intervention/treatment/program	Describe the focus or goal of the intervention/treatment/program
		Describe the type of rehabilitation intervention/treatment/program, how it was delivered, the length or frequency of the intervention/treatment/program, and the setting of rehabilitation
		Note the theories or principles of care that are guiding the rehabilitation studied in the article
		Note if/describe how the intervention/treatment/program acknowledged and/or accounted for intersecting social identities and vulnerabilities and CJS involvement at the time of the intervention
	Rehabilitation team	List the healthcare providers/professional disciplines that were involved in the intervention/treatment/program or rehabilitation process
		Note if the rehabilitation team collaborates with or have access to other providers/disciplines not listed in Table 1
	Outcome	Describe the outcome of rehabilitation
		Note any outcome(s) relevant to intersectionality
	Barriers	Describe any stated barriers to rehabilitation for individuals with TBI who intersect with the CJS
	Facilitators	Describe any stated facilitators to rehabilitation for individuals with TBI who intersect with the CJS
	Gaps	Describe any stated gaps in research on rehabilitation for individuals with TBI who intersect with the CJS

### Collating, summarizing, and reporting the results

We will follow a three-part process, as suggested by Levac et al. [33]. The analysis of the data will include quantitative descriptive numerical summaries of (a) study design, (b) study sample, (c) rehabilitation program or intervention, team member(s), and outcomes by study design, and (d) TBI-related barriers, facilitators, and gaps. Qualitative content analytic techniques will also be used to identify themes or categories in relation to the research question [43]. We will also assess the internal validity of the included studies using the Study Quality Assessment Tools designed by methodologists from the National Institutes of Health’s National Heart, Lung, and Blood Institute and the Research Triangle Institute International [44]. No studies will be excluded from the scoping review based on the quality assessment; however, results of this critical appraisal will be used to inform the interpretation of results from this review. Overall, findings from the analysis will be reported in relation to the research question. In particular, we will consider implications for opportunities to integrate rehabilitation for individuals with TBI who intersect with all parts of the CJS and recommendations for future research.

### Consultation

We will engage stakeholders to identify further relevant literature and for feedback on our findings. This will aid us in identifying literature not captured in the above search strategy, particularly unpublished literature. Stakeholders for this scoping review may include front-line staff and service providers in the CJS and brain injury sectors; health administrators, decision-makers, and policy-makers; health professionals who provide care for individuals with TBI and/or individuals who have intersected with the CJS; researchers and trainees who conduct research on rehabilitation, TBI, and the CJS; and caregivers or family members of individuals with lived experience of TBI and/or CJS intersection. Specifically, they will be engaged through an established Program Advisory Committee (PAC) of the Traumatic Brain Injury in Underserved Populations Research Program [45, 46]. The PAC collaborates with the research team on research and knowledge dissemination activities and currently meets every three to four months per year. Findings will be presented at a PAC meeting and the PAC members’ feedback will be documented and integrated into the scoping review.

### Ethics and dissemination

Only published and publicly available data will be analyzed and thus, research ethics approval will not be required. We will publish the scoping review in a peer-reviewed journal and present findings at scientific conferences and to stakeholders.

### Strengths and limitations

A strength of this protocol is the consideration of literature addressing all parts of the CJS for the scoping review. Since individuals in the correctional system must have proceeded through policing and courts, and will proceed to parole, investigating all parts of the CJS provides a comprehensive summary of existing rehabilitation for individuals with TBI who intersect with the CJS. In addition, we will explicitly identify sex, gender, and intersecting identities, which not only drive power relations and inequities, but also intersect with other forms of inequality [47]. This protocol also considers experiences of homelessness, as TBI is associated with both the CJS and homelessness [48]. For example, a 2019 systematic review of primarily North American research found that homeless and marginally housed individuals have a lifetime TBI prevalence of 2.5 to 4.0 times greater than the general population [48]. In addition, a Canadian study of 1,181 homeless and vulnerably housed individuals found that the odds of being incarcerated or arrested were 1.8 times greater in those with TBI [49]. The explicit consideration of intersecting identities and experiences provide us with a powerful tool with which to understand and analyze the occurrence and effect of intersecting identities and related systems of power and inequality [50].

While we will critically appraise the internal validity of the included studies, no articles will be excluded based on the quality assessment. As such, a limitation of this review is that it will not consider the effectiveness of the rehabilitation interventions or programs identified from this review. However, we believe the scoping review to explore the extent to which rehabilitation, including the types of rehabilitation interventions, are used by or available to individuals with TBI who intersect with the CJS, is an important first step to understand the current literature and to identify areas for future research. We further recognize that only articles that describe all three concepts (CJS, rehabilitation, and TBI) will be included in the review. As such, the review will miss articles that do not explicitly specify their sample to including individuals with TBI, as well as studies on rehabilitation in the CJS among individuals experiencing cognitive impairments without screening for TBI.

## Conclusion

TBI is a significant cause of both death and disability worldwide and is disproportionately prevalent among individuals who intersect with the CJS [2]. While rehabilitation holds the potential to address individual and social impacts of TBI-related disability [22–26], there are few reviews on rehabilitation among individuals with TBI who intersect with the CJS. Concurrently, to the best of our knowledge, there is currently no review that explores rehabilitation among individuals with TBI who intersect with all parts of the CJS. This protocol documents a transparent approach to addressing this gap in knowledge. Additionally, this protocol explicitly outlines the charting of data considering intersecting identities (e.g., race, ethnicity, disability, and experience with violence and homelessness); this will aid in identifying inequities experienced by, and gaps in knowledge of, diverse individuals with TBI who intersect with the CJS. Finally, findings from the scoping review will inform opportunities to integrate rehabilitation for individuals with TBI who intersect with the CJS and identify opportunities for future research.

## Supporting information

S1 Checklist (PDF)

S1 File Search description and strategy. (PDF)

## References

[1] Dewan M.C., et al., Estimating the global incidence of traumatic brain injury. J Neurosurg, 2018: p. 1–18. doi: 10.3171/2017.10.JNS17352 29701556

[2] Farrer T.J. and Hedges D.W., Prevalence of traumatic brain injury in incarcerated groups compared to the general population: a meta-analysis. Prog Neuropsychopharmacol Biol Psychiatry, 2011. 35(2): p. 390–4. doi: 10.1016/j.pnpbp.2011.01.007 21238529

[3] Shiroma E.J., Ferguson P.L., and Pickelsimer E.E., Prevalence of traumatic brain injury in an offender population: a meta-analysis. J Correct Health Care, 2010. 16(2): p. 147–59. doi: 10.1177/1078345809356538 20339132

[4] Durand E., et al., History of traumatic brain injury in prison populations: A systematic review. Ann Phys Rehabil Med, 2017. 60(2): p. 95–101. doi: 10.1016/j.rehab.2017.02.003 28359842

[5] Hughes N., et al., The prevalence of traumatic brain injury among young offenders in custody: a systematic review. J Head Trauma Rehabil, 2015. 30(2): p. 94–105. doi: 10.1097/HTR.0000000000000124 25734840

[6] Moynan C.R. and McMillan T.M., Prevalence of Head Injury and Associated Disability in Prison Populations: A Systematic Review. J Head Trauma Rehabil, 2018. 33(4): p. 275–282. doi: 10.1097/HTR.0000000000000354 29084104

[7] McGinley A. and McMillan T., The prevalence, characteristics, and impact of head injury in female prisoners: a systematic PRISMA review. Brain Inj, 2019. 33(13–14): p. 1581–1591. doi: 10.1080/02699052.2019.1658223 31456433

[8] Williams H., Repairing shattered lives: Brain injury and its implications for criminal justice in Transition to Adulthood. 2012, Barrow Cadbury Trust on behalf of the Transition to Adulthood Alliance.

[9] Williams W.H., et al., Traumatic brain injury: a potential cause of violent crime? Lancet Psychiatry, 2018. 5(10): p. 836–844. doi: 10.1016/S2215-0366(18)30062-2 29496587 PMC6171742

[10] Matheson F.I., et al., Association between traumatic brain injury and prison charges: a population-based cohort study. Brain Injury, 2020. 34(6): p. 757–765. doi: 10.1080/02699052.2020.1753114 32324431

[11] Centers for Disease Control and Prevention, U.S.D.o.H.a.H.S., Traumatic Brain Injury in Prisons and Jails: An Unrecognized Problem. 2007.

[12] Ray B. and Richardson N.J., Traumatic Brain Injury and Recidivism Among Returning Inmates. Criminal Justice and Behavior, 2017. 44(3): p. 472–486.

[13] Williams W.H., et al., Traumatic brain injury in a prison population: Prevalence and risk for re-offending. Brain Injury, 2010. 24(10): p. 1184–1188. doi: 10.3109/02699052.2010.495697 20642322

[14] Schwartz J.A., et al., Changes in Jail Admissions Before and After Traumatic Brain Injury. Journal of Quantitative Criminology, 2021.

[15] Schwartz J.A., A Longitudinal Assessment of Head Injuries as a Source of Acquired Neuropsychological Deficits and the Implications for Criminal Persistence. Justice Quarterly, 2021. 38(2): p. 196–223.

[16] Hart T., Sander A. Memory and Moderate to Severe Traumatic Brain Injury 2016; Available from: https://msktc.org/tbi/factsheets/Memory-And-Traumatic-Brain-Injury.

[17] Wszalek J.A. and Turkstra L.S., Comprehension of Legal Language by Adults With and Without Traumatic Brain Injury. J Head Trauma Rehabil, 2019. 34(3): p. E55–e63. doi: 10.1097/HTR.0000000000000434 30169438 PMC6395580

[18] McKinlay A. and Albicini M., Prevalence of traumatic brain injury and mental health problems among individuals within the criminal justice system. Concussion, 2016. 1(4): p. Cnc25. doi: 10.2217/cnc-2016-0011 30202566 PMC6093757

[19] McIsaac K.E., et al., Association between traumatic brain injury and incarceration: a population-based cohort study. CMAJ Open, 2016. 4(4): p. E746–e753. doi: 10.9778/cmajo.20160072 28018890 PMC5173464

[20] World Health Organization. Rehabilitation. 2021 November 10, 2021; Available from: https://www.who.int/news-room/fact-sheets/detail/rehabilitation.

[21] Lamontagne M-E T.C., Kagan C, Bayley M, Swaine B, Marshall S, et al. INESSS-ONF clinical practice guidelines for the rehabilitation of adults having sustained a moderate-to-severe TBI. 2017 June 23, 2020; Available from: https://braininjuryguidelines.org/modtosevere/.

[22] Ross E.H. and Hoaken P.N.S., Correctional Remediation Meets Neuropsychological Rehabilitation: How Brain Injury and Schizophrenia Research Can Improve Offender Programming. Criminal Justice and Behavior, 2010. 37(6): p. 656–677.

[23] Iaccarino MA B.S., Zafonte R, Chapter 26—Rehabilitation after traumatic brain injury, in Handbook of Clinical Neurology, Grafman J S.A., Editor. 2015, Elsevier. p. 411–422. 10.1016/B978-0-444-52892-6.00026-X 25702231

[24] Fadyl J.K. and McPherson K.M., Approaches to vocational rehabilitation after traumatic brain injury: a review of the evidence. J Head Trauma Rehabil, 2009. 24(3): p. 195–212. doi: 10.1097/HTR.0b013e3181a0d458 19461367

[25] Stephens J.A., Williamson K.N., and Berryhill M.E., Cognitive Rehabilitation After Traumatic Brain Injury: A Reference for Occupational Therapists. OTJR (Thorofare N J), 2015. 35(1): p. 5–22. doi: 10.1177/1539449214561765 26623474 PMC6730543

[26] Kim H. and Colantonio A., Effectiveness of rehabilitation in enhancing community integration after acute traumatic brain injury: a systematic review. Am J Occup Ther, 2010. 64(5): p. 709–19. doi: 10.5014/ajot.2010.09188 21073101

[27] de Geus E.Q.J., et al., Acquired Brain Injury and Interventions in the Offender Population: A Systematic Review. Frontiers in psychiatry, 2021. 12: p. 658328–658328. doi: 10.3389/fpsyt.2021.658328 34025480 PMC8138134

[28] McDonald B.C., Flashman L.A., and Saykin A.J., Executive dysfunction following traumatic brain injury: neural substrates and treatment strategies. NeuroRehabilitation, 2002. 17(4): p. 333–44. 12547981

[29] Correctional Service Canada. Section 3—Federal Corrections and the Criminal Justice System. 2007 April 17, 2008 [cited 2022 May 27]; Available from: https://www.csc-scc.gc.ca/text/pblct/sb-go/03-eng.shtml.

[30] Crown Prosecution Service. The Criminal Justice System | The Crown Prosecution Service. 2017 [cited 2022 May 27]; Available from: https://www.cps.gov.uk/about-cps/criminal-justice-system.

[31] Bureau of Justice Statistics. The Justice System. 2021 [cited 2022 May 27]; Available from: https://bjs.ojp.gov/justice-system.

[32] Arksey H. and O’Malley L., Scoping studies: towards a methodological framework. International Journal of Social Research Methodology, 2005. 8(1): p. 19–32.

[33] Levac D., Colquhoun H., and O’Brien K.K., Scoping studies: advancing the methodology. Implement Sci, 2010. 5: p. 69. doi: 10.1186/1748-5908-5-69 20854677 PMC2954944

[34] Tricco A.C., et al., PRISMA Extension for Scoping Reviews (PRISMA-ScR): Checklist and Explanation. Annals of Internal Medicine, 2018. 169(7): p. 467–473. doi: 10.7326/M18-0850 30178033

[35] Menon D.K., et al., Position statement: definition of traumatic brain injury. Arch Phys Med Rehabil, 2010. 91(11): p. 1637–40. doi: 10.1016/j.apmr.2010.05.017 21044706

[36] Reed N Z.R., Dawson J, Ledoux A, Provvidenza C, Paniccia M, et al. Guidelines for Diagnosing and Managing Pediatric Concussion. Available from: www.braininjuryguidelines.org.

[37] Lee C., et al., A systematic integrative review of programmes addressing the social care needs of older prisoners. Health & Justice, 2019. 7(1): p. 9. doi: 10.1186/s40352-019-0090-0 31134392 PMC6717991

[38] Chan V., et al., Protocol for a scoping review on rehabilitation among individuals who experience homelessness and traumatic brain injury. BMJ Open, 2021. 11(11): p. e052942. doi: 10.1136/bmjopen-2021-052942 34740933 PMC8573664

[39] Rethlefsen M.L., et al., PRISMA-S: an extension to the PRISMA Statement for Reporting Literature Searches in Systematic Reviews. Syst Rev, 2021. 10(1): p. 39. doi: 10.1186/s13643-020-01542-z 33499930 PMC7839230

[40] Team, T.E., EndNote. 2013, Clarivate Analytics: Philadelphia, PA.

[41] Veritas Health Innovation, Covidence systematic review software. Melbourne, Australia.

[42] Women and Gender Equality Canada. What is Gender-based Analysis Plus. 2021 April 14, 2021; Available from: https://women-gender-equality.canada.ca/en/gender-based-analysis-plus/what-gender-based-analysis-plus.html.

[43] Hsieh H.-F. and Shannon S.E., Three Approaches to Qualitative Content Analysis. Qualitative Health Research, 2005. 15(9): p. 1277–1288. doi: 10.1177/1049732305276687 16204405

[44] National Heart, L., and Blood Institute,. Background: Development and Use of Study Quality Assessment Tools. Available from: https://www.nhlbi.nih.gov/node/80102.

[45] Colantonio A., Chan V., Traumatic Brain Injury in Underserved Populations. 2021, Ontario Ministry of Health and Long-Term Care.

[46] Colantonio A., Canada Research Chair Tier 1—Traumatic brain injury in underserved populations. 2020, Canada Research Chairs Programs.

[47] Theobald S., et al., The importance of gender analysis in research for health systems strengthening. Health Policy Plan, 2017. 32(suppl_5): p. v1–v3. doi: 10.1093/heapol/czx163 29244107 PMC5886229

[48] Stubbs J.L., et al., Traumatic brain injury in homeless and marginally housed individuals: a systematic review and meta-analysis. The Lancet Public Health, 2020. 5(1): p. e19–e32. doi: 10.1016/S2468-2667(19)30188-4 31806487

[49] To M.J., et al., Healthcare Utilization, Legal Incidents, and Victimization Following Traumatic Brain Injury in Homeless and Vulnerably Housed Individuals: A Prospective Cohort Study. J Head Trauma Rehabil, 2015. 30(4): p. 270–6. doi: 10.1097/HTR.0000000000000044 24651000

[50] Abrams J.A., et al., Considerations for employing intersectionality in qualitative health research. Soc Sci Med, 2020. 258: p. 113138. doi: 10.1016/j.socscimed.2020.113138 32574889 PMC7363589

