# Supplementary material for: Protocol for a scoping review on rehabilitation among individuals with traumatic brain injury who intersect with the criminal justice system
Source: PLoS One. 2022 Jun 30;17(6):e0269696. doi: 10.1371/journal.pone.0269696 (PMC9246198; doi:10.1371/journal.pone.0269696)
Supplement: S1 File — (PDF) [file pone.0269696.s002.pdf]

Supplementary File 1

Search Description
This strategy has been developed in Medline, and will be translated to other databases. It uses the following concepts:

- Concept A (lines 1-24): Criminal Justice System
- Concept B (lines 25-64): Rehabilitation
- Concept C (lines 65-77): TBI or cognitive impairment

The search conducted is: (A + B + C). This strategy will be used in all searched databases. Searches will be limited to human studies when possible. No date or language limits will be applied.

Search Strategy

Database: Ovid MEDLINE(R) ALL <1946 to July 02, 2021>

1 exp PRISONS/
2 exp PRISONERS/
3 exp CRIMINALS/
4 Criminal Law/ or Jurisprudence/
5 Judicial Role/
6 (jurisprudenc* or ligitat*).tw,kf.
7 (legal adj (system? or servic*)).tw,kf.
8 (prisoner* or prison? or imprison*).tw,kf.
9 (inmate* or convict* or criminal* or offender?).tw,kf.
10 (correctional adj2 (setting? or service? or units or unit or facility or facilities or institution* or centre* or center*)).tw,kf.
11 (penal adj2 (setting? or service? or units or unit or facility or facilities or institution* or centre* or center*)).tw,kf.
12 (jail* or penitentiar* or gaol*).tw,kf.
13 incarcerat*.tw,kf.
14 (detain* or detention?).tw,kf.
15 parole?.tw,kf.
16 probation*.tw,kf.
17 felon*.tw,kf.
18 Police/
19 (police or policing).tw,kf.
20 law enforce*.tw,kf.
21 forensic*.tw,kf.
22 forensic psychiatry/ or "commitment of mentally ill"/ or insanity defense/
23 (correctional or forensic).jw.
24 or/1-23
25 "Physical and Rehabilitation Medicine"/

26 exp rehabilitation/
27 rehab*.tw,kf,jw.
28 telerehab*.tw,kf,jw.
29 neurorehab*.tw,kf,jw.
30 rh.fs.
31 (physiatrist? or physiatry).tw,kf.
32 Rehabilitation Centers/
33 occupational therapy/
34 (occupational adj therap*).tw,kf,jw.
35 physical therapy specialty/
36 (physical adj therap*).tw,kf,jw.
37 physiotherap*.tw,kf,jw.
38 physio-therapist*.tw,kf,jw.
39 Speech-Language Pathology/
40 (speech adj2 (therap* or patholog*)).tw,kf,jw.
41 Neuropsychology/
42 Neuropsycholog*.tw,kf,jw.
43 Nutritionists/
44 (Nutritionist? or Dietician?).tw,kf,jw.
45 (therap* adj recreation*).tw,kf,jw.
46 child life specialist?.tw,kf.
47 play therapy/
48 (play adj therap*).tw,kf.
49 Respite Care/
50 respite.tw,kf.
51 Case Managers/
52 Case Management/
53 case manag*.tw,kf.
54 exp Social Work/
55 social work*.tw,kf,jw.
56 Forensic Nursing/
57 (nurse? or nursing).tw,kf,jw.
58 Community Integration/
59 (integrat* or reintegrat* or re-integrat* or reentry or re-entry or resettle* or re-settle*).tw,kf.
60 Aftercare/
61 (Aftercare or "after care").tw,kf.
62 Transitional Care/
63 "transitional care".tw,kf.
64 or/25-63
65 exp Brain Injuries/
66 exp Brain Injuries, Traumatic/
67 exp Brain Concussion/

68 Craniocerebral Trauma/
69 tbi*2.tw,kf.
70 mtbi*2.tw,kf.
71 concuss*.tw,kf.
72 postconcuss*.tw,kf.
73 ((head* or brain* or cerebr* or crani* or skull* or intracran*) adj2 (injur* or trauma* or damag* or wound* or swell* or oedema* or edema* or fracture* or contusion* or pressur*)).tw,kf,jw.
74 ((brain* or cerebr* or intracerebr* or crani* or intracran* or head* or subdural* or epidural* or extradural*) adj (haematoma* or hematoma* or hemorrhag* or haemorrhag* or bleed*)).tw,kf.
75 exp cognition disorders/
76 ((cogniti* or neurocogniti*) adj2 (impair* or dysfunction* or disorder* or declin*)).tw,kf.
77 or/65-76
78 24 and 64
79 24 and 77
80 24 and 64 and 77
81 80 not (exp animals/ not humans.sh.)

Grey Literature

Reports from the following brain injury, criminal justice system, and rehabilitation organizations will be searched:

- ABI Justice
- Acquired Brain Injury Ireland
- American Academy of Physical Medicine and Rehabilitation
- Australian Capital Territory Corrective Services
- Barrow Cadbury Trust
- Brain and Spine Foundation
- Brain Injury Association of America
- Brain Injury Australia (BIA)
- Brain Injury Canada+ Provincial and local brain injury associations identified therein
- Brain Injury New Zealand
- Brain Injury Rehabilitation Trust (BIRT)
- Brain Research UK
- Brain Trauma Foundation
- British Society of Rehabilitation Medicine
- Canadian Association of Elizabeth Fry
- Canadian Association of Physical Medicine and Rehab
- Canadian Mental Health Association

- Centre for Crime and Justice Studies
- Cheshire and Merseyside Rehabilitation Network
- Child Brain Injury Trust
- Concussions Ontario
- Connectivity Traumatic Brain Injury Australia
- Correctional Service Canada
- Criminal Justice Alliance
- Department of Corrections (New Zealand)
- Disability Federation of Ireland
- Disability Services USAGov
- Enable Ireland
- European Brain Injury Society
- Federal Bureau of Prisons
- GTA Rehab Network
- Headway
- Headway : Brain Injury Auckland New Zealand
- Headway Ireland
- Her Majesty's Prison and Probation Service
- Human Services and Justice Coordinating Committee
- International Brain Injury Association
- Irish Penal Reform Trust
- Irish Prison Service
- John Howard Society
- John Howard Society of Ontario
- Justice (UK charity)
- JustSpeak
- Kessler Foundation
- Law and Justice Foundation of NSW
- MacArthur Foundation
- March of Dimes Canada
- Mental Health Commission of Canada
- Mental Health Foundation (UK)
- Mental Health Ireland
- Ministry of Health Disability Services
- National Institute of Mental Health (NIMH)
- National Mental Health Consumer and Carer Forum
- New South Wales Corrective Services
- New Zealand Parole Board
- Northern Territory Correctional Services
- Office for Disability Issues
- Office of Disability Employment Policy (ODEP)

- Parole Board (Ireland)
- Parole Board of Canada
- People with Disabilities Act
- Probation Officers Association of Ontario
- PSR RPS Canada
- Queensland Corrective Services
- Rehabilitation Services Administration (RSA)
- SameYou
- South Australia Department for Correctional Services
- Synapse
- Tasmania Corrective Services
- The Center on Brain Injury Research & Training
- The Disabilities Trust
- The Parole Board (UK)
- The Sentencing Project
- Toronto ABI Network
- Toronto Rehabilitation Institute
- Traumatic Brain Injury Center of Excellence
- U.S. Department of Justice
- United Kingdom Acquired Brain Injury Forum
- Victoria Corrections Prisons and Parole
- Western Australia Department of Corrective Services
- Women with Disabilities Victoria
